# Development of a DNA Metabarcoding Method for the Identification of Bivalve Species in Seafood Products

**DOI:** 10.3390/foods10112618

**Published:** 2021-10-28

**Authors:** Kristina Gense, Verena Peterseil, Alma Licina, Martin Wagner, Margit Cichna-Markl, Stefanie Dobrovolny, Rupert Hochegger

**Affiliations:** 1 Austrian Competence Centre for Feed and Food Quality, Safety and Innovation, FFoQSI GmbH, Technopark 1, 3430 Tulln an der Donau, Austria; kristina.gense@ffoqsi.at (K.G.); martin.wagner@ffoqsi.at (M.W.); 2 Austrian Agency for Health and Food Safety (AGES), Institute for Food Safety, Department of Molecular Biology and Microbiology, Spargelfeldstr. 191, 1220 Vienna, Austria; verena.peterseil@ages.at (V.P.); stefanie.dobrovolny@ages.at (S.D.); 3 LVA GmbH, Magdeburggasse 10, 3400 Klosterneuburg, Austria; alma.licina@lva.at; 4 Department for Farm Animals and Veterinary Public Health, Institute of Milk Hygiene, University of Veterinary Medicine, Veterinärplatz 1, 1210 Vienna, Austria; 5 Department of Analytical Chemistry, Faculty of Chemistry, University of Vienna, Währinger Straße 38, 1090 Vienna, Austria; margit.cichna@univie.ac.at

**Keywords:** DNA metabarcoding, next generation sequencing, food authentication, bivalves, Mytilidae, Pectinidae, Ostreidae, species identification, mitochondrial 16S rDNA, seafood

## Abstract

The production of bivalve species has been increasing in the last decades. In spite of strict requirements for species declaration, incorrect labelling of bivalve products has repeatedly been detected. We present a DNA metabarcoding method allowing the identification of bivalve species belonging to the bivalve families Mytilidae (mussels), Pectinidae (scallops), and Ostreidae (oysters) in foodstuffs. The method, developed on Illumina instruments, targets a 150 bp fragment of mitochondrial 16S rDNA. We designed seven primers (three primers for mussel species, two primers for scallop species and a primer pair for oyster species) and combined them in a triplex PCR assay. In each of eleven reference samples, the bivalve species was identified correctly. In ten DNA extract mixtures, not only the main component (97.0–98.0%) but also the minor components (0.5–1.5%) were detected correctly, with only a few exceptions. The DNA metabarcoding method was found to be applicable to complex and processed foodstuffs, allowing the identification of bivalves in, e.g., marinated form, in sauces, in seafood mixes and even in instant noodle seafood. The method is highly suitable for food authentication in routine analysis, in particular in combination with a DNA metabarcoding method for mammalian and poultry species published recently.

## 1. Introduction

Bivalves, a class of molluscs, are distributed worldwide. Due to their high content of essential nutrients, their production has steadily been increased over the last three decades [1,2,3,4,5]. Mytilidae (mussels), Pectinidae (scallops), and Ostreidae (oysters) are the most important bivalve families for human consumption. Each of these bivalve families is divided into several genera comprising a high number of species [6]. In 2019, 1.03 million tons of mussels, scallops, and oysters were caught in nature and 10.25 million tons were cultivated in aquaculture, earning a profit of millions of US dollars [7].

In the EU, international and national regulations exist to ensure legal trade in seafood and seafood products. The EU directive 1379/2013 regulates market organization of fishery and aquaculture products, including correct declaration of seafood [8]. To comply with legal regulations, labels must include both the local trade name in the official language(s) and the correct scientific Latin name [8,9]. Correct labelling of seafood products is important for traceability issues, protection of endangered species, mitigation of illegal fishing, and for individual reasons of end consumers [10,11]. Regardless of clear and strict requirements for species declaration, incorrect labelling of bivalve products has repeatedly been detected in Europe [12,13,14,15,16,17]. In German and Swiss studies, more than half of the products declared to contain “Jakobsmuschel” (or “Jacobsmuschel“) were labelled incorrectly [15,18,19]. Although the German name “Jakobsmuschel” (or “Jacobsmuschel“) may only be used for scallop species belonging to the genus *Pecten*, species of other genera (particularly *Placopecten* and *Mizuhopecten*) were identified in these products.

For authentication of seafood products, laboratories may choose from a variety of methodologies. In the case of bivalves, morphological characteristics such as shell, color, and size may allow correct species classification. However, after shell removal or mechanical processing, classification by morphology may be hampered or even be impossible [16,20]. Recently, matrix-assisted laser desorption ionization time of flight mass spectrometry (MALDI-TOF MS) has been shown to be suitable for accurate species identification of scallops [19]. However, since MALDI-TOF MS instruments are rather expensive and do not allow high-throughput analysis, this methodology is less applicable for routine analyses.

To date, DNA-based methods are considered most suitable for the identification of seafood species, even in highly processed food products [21,22,23]. Due to its high copy number and robustness, mitochondrial DNA (mtDNA) is frequently preferred over genomic DNA [24,25]. The mtDNA regions most commonly used for species identification are cytochrome c oxidase subunit I (COI), cytochrome b (cyt b), and 16S ribosomal DNA (16S rDNA) [15,26,27,28,29,30,31,32,33]. Compared to other seafood, e.g., fish, crustaceans, and cephalopods, (real-time) polymerase chain reaction (PCR) assays for bivalve species are limited in number [18,32,34,35,36,37,38,39,40,41]. The disadvantage of (real-time) PCR is that for each target species, a specific primer (probe) system is required [18,31,33,36,39,40,41,42,43].

A powerful alternative is DNA barcoding, aiming at detecting a broader range of species by using universal primer systems [22,26,34,44]. DNA barcodes commonly contain conserved regions at both ends, serving as binding sites for universal primers, and a variable part in between the primer binding sites, for differentiation between the species of interest [34,45]. DNA barcodes of approximately 600 base pairs (bp) in length have been found to be suitable for the analysis of highly processed food products [22,26,27,34,44,46,47,48]. In conventional DNA barcoding, PCR products obtained by amplifying the selected DNA barcode region are then subjected to Sanger sequencing [22,34,44,49,50]. However, sample throughput of Sanger sequencing is limited since samples are sequenced one by one. A much more efficient approach is to combine DNA barcoding with next-generation sequencing (NGS) technologies [22,26,34]. So-called DNA metabarcoding allows the identification of multiple species in multiple food samples in one and the same sequencing run [45,46,51,52,53,54]. The suitability of DNA metabarcoding for the analysis of ultra-processed food products has already been demonstrated, e.g., for the detection of mammals in sausages or insects in bars [47,48].

In this study, we present a DNA metabarcoding method allowing the differentiation between species from three bivalve families, Pectinidae, Ostreidae, and Mytilidae*,* in raw and processed food products to detect food adulteration. The method was developed on the Illumina MiSeq^®^ (San Diego, CA, USA) and iSeq^®^ (San Diego, CA, USA) platforms due to their low error rates compared to other NGS platforms [55].

## 2. Materials and Methods

### 2.1. Sample Collection and Storage

A total of 86 commercial food products were collected from regional supermarkets, fish markets, and delicacy shops in Austria from summer 2018 until winter 2020 (Supplementary Table S1). Samples were either fresh, deep-frozen, or in processed condition. Each sample was given a specific ID number, with the letter “O” referring to oysters, “S” to scallops, “M” to mussels, and “Mi” to mixed-species seafood. Samples were stored at −20 °C until DNA extraction.

Eleven out of the 86 samples (“reference samples”), comprising three mussel, six scallop, and two oyster species (see Table 1), were used for method development. Identity of bivalve species in these reference samples (samples M12, M13 and M27 for mussels; samples S42, S46, S47, S49, S50, and S55 for scallops; samples O2 and O3 for oysters; Supplementary Table S1) was verified by subjecting DNA extracts to Sanger sequencing (Microsynth, Balgach, Switzerland) and matching the sequences against the public databases provided by the National Center for Biotechnology Information (NCBI, Bethesda, MD, USA). For Sanger sequencing, the forward and reverse primers listed in Table 2 were used.

### 2.2. DNA Extraction and Quantification

Raw material was cut into smaller pieces or homogenized. To 2.0 g of each sample, 10 mL of a hexadecyltrimethylammonium bromide (CTAB) buffer was added. After addition of 80 µL proteinase K, the mixture was incubated on an Intelli-Mixer^TM^ RM2 (LTF Labortechnik, Wasserburg, Germany) overnight at 50 °C.

For DNA isolation, a commercial kit (Maxwell^®^ 16 FFS Nucleic Acid Extraction System Custom-Kit, Promega, Madison, WI, USA) was used according to the manufacturer’s instructions. DNA concentration was determined fluorometrically (Qubit^®^ 2.0 fluorometer, Thermo Fisher Scientific, Waltham, MA, USA). For higher concentrations, the Qubit^®^ dsDNA broad range assay kit (2 to 1000 ng) was used, and for lower concentrations, the Qubit^®^ dsDNA high-sensitivity assay kit (0.2 to 100 ng) was used. DNA purity was assessed from the ratio of the absorbance at 260 and 280 nm (QIAxpert spectrophotometer, software version 2.2.0.21, Qiagen, Hilden, Germany). DNA extracts were stored at −20 °C until further use.

### 2.3. DNA Extract Mixtures

Ternary DNA extract mixtures were prepared by mixing DNA extracts (DNA concentration 5 ng/µL) from *Pecten* spp., *Magallana gigas* and *Mytilus galloprovincialis*, representing the three bivalve families Pectinidae, Ostreidae, and Mytilidae, respectively. Individual DNA extracts were mixed in a ratio of 98.0:1.5:0.5 (*v*/*v*/*v*).

In addition, DNA extract mixtures consisting of DNA from species belonging to one bivalve family were prepared. In these mixtures, DNA from one species was present as the main component, DNA from the other species as minor components (1.0% each). Since only two oyster species were available, the DNA extract mixture representing the bivalve family Ostreidae contained the closely related scallop (*Placopecten magellanicus*) as a major component (98.0%) and DNA from the two oyster species as minor components (1.0% each).

In addition to mixtures consisting of DNA from bivalve species only, a DNA extract mixture containing another mollusc species was prepared. DNA extract from a squid species (*Sepiella inermis*) was chosen as the main component (97.0%) and DNA from the bivalve species *Placopecten magellanicus*, *Ostrea edulis* and *Perna canaliculus* was present as minor components (1.0% each).

### 2.4. Reference Sequences

A 150 bp fragment of the mitochondrial 16S rDNA gene was used as a DNA barcode. Reference sequences for commonly consumed bivalve species and some exotic seafood species, that are permitted for consumption in Austria (“Codex Alimentarius Austriacus” chapter B35, [56]), were downloaded from the NCBI databases (Supplementary Table S2) by using CLC Genomics Workbench software (version 10.1.1, Qiagen, Hilden, Germany). If available, complete reference sequences from the RefSeq database were preferentially downloaded due to their reliability. In case complete reference sequences were not available, all DNA sequences of the mitochondrial 16S rDNA available for one and the same species, submitted by individual scientists, were aligned and checked for similarity and unidentified nucleotides. Subsequently, the DNA sequence with the highest quality (e.g., without unknown nucleotides, full-length of the DNA barcode) was chosen as a reference sequence.

### 2.5. Primer Systems

Primers were designed manually on a multiple DNA sequence alignment of the mitochondrial 16S rDNA of approximately 90 bivalve species using the CLC Genomics Workbench software (version 10.1.1, Qiagen, Hilden, Germany). The designed primers were checked for their physical and structural properties (e.g., formation of dimers, secondary structure, annealing temperature) using Oligo Calc, the OligoAnalyzer Tool provided by Integrated DNA Technologies (IDT, Coralville, IA, USA) and the online product descriptions from TIB Molbiol (Berlin, Germany). The primers, listed in Table 2, were synthesized by TIB Molbiol. Table 2 also shows the Illumina overhang adapter sequences which were linked to the target-specific primers.

All in-house-designed primers were tested in real-time PCR with DNA extracted from the eleven reference samples. During optimization, the following PCR conditions/parameters were kept constant and applied as published previously: DNA input amount of 12.5 ng, ‘ready-to-use’ HotStarTaq Master Mix Kit, annealing temperature (62 °C), 25 cycles [47]. Only one variable, the addition of magnesium chloride solution, was modified (addition of 1.5 or 3 mM MgCl_2_). Real-time PCR reactions were carried out using a fluorescent intercalating dye (EvaGreen^®^ (20x in water)) in strip tubes or in 96-well plates, depending on the thermocycler used, the Rotor-Gene Q (Qiagen, Hilden, Germany) or the LightCycler^®^ 480 System (Roche, Penzberg, Germany), respectively. The total volume of the PCR reactions was 25 µL, consisting of 22.5 µL reaction mix and 2.5 µL of template DNA (diluted DNA samples (5 ng/µL)) or water as negative control. In the reaction mix, the HotStarTaq Master Mix Kit (Qiagen, Hilden, Germany) was used at a final concentration of 1x and the final concentration of primers was 0.2 µM, except the forward primer for mussels (0.4 µM). PCR cycling conditions were 15 min initial denaturation at 95 °C, 25 cycles at 95 °C, 62 °C and 72 °C for 30 s each, and a final elongation for 10 min at 72 °C. The primer pairs for mussels, scallops, and oysters with and without Illumina overhang adapter sequences were first used in singleplex PCR assays. Then, the seven primers (three forward and four reverse primers) listed in Table 2 were combined in a triplex assay. The identity of the PCR products was confirmed by melting curve analysis and/or agarose gel electrophoresis.

### 2.6. Library Preparation and NGS

In general, samples were sequenced by using either the MiSeq^®^ or the iSeq^®^ platform (Illumina, San Diego, CA, USA). DNA extracts were diluted to a DNA concentration of 5 ng/μL. Extracts with a DNA concentration < 5 ng/μL were used undiluted.

DNA library preparation was performed according to Dobrovolny et al. [47] with minor modifications (excess of MgCl_2_, final concentration 3 mM; average library size: 278 bp; diluted libraries of the iSeq^®^ system were denatured automatically on the instrument).

For the MiSeq^®^ and iSeq^®^ platform, the DNA library was adjusted to 4 and 1 nM, respectively, with 10 mM Tris-HCl, pH 8.6. After pooling individual DNA libraries (5 µL MiSeq^®^, 7 µL iSeq^®^), the DNA concentration was determined using Qubit^®^ 2.0 fluorimeter.

All sequencing runs were performed using either the MiSeq^®^ Reagent Kit v2 (300-cycles) or the iSeq^®^ 100 i1 Reagent v2 (300-cycles) with a final loading concentration of 8 pM. The pooled DNA libraries contained a 5% PhiX spike-in.

Reference samples were sequenced in six replicates (three sequencing runs, two replicates per run), while DNA extract mixtures were sequenced in nine replicates (three sequencing runs, three replicates per run). Commercial food products were sequenced in triplicates (three sequencing runs, one replicate per run) and food products were sequenced at least once by using either the MiSeq^®^ or the iSeq^®^ platform.

### 2.7. NGS Data Analysis Using Galaxy

After paired-end sequencing, the resulting FastQ files, generated by the instrument control software, were used as input for data analysis. The sequencing output in FastQ format was then processed with an analysis pipeline as described previously by using Galaxy (version 19.01) [47]. The published amplicon analysis workflow was modified as follows: the target-specific primers were trimmed from both ends using the tool Cutadapt and reads were not clustered into Operational Taxonomic Units (OTUs) [57]. Completely identical sequences were collapsed into a single representative sequence with the tool Dereplicate to minimize the number of reads, and then compared against a customized database for bivalves (Supplementary Table S2) using BLASTn [58].

## 3. Results and Discussion

### 3.1. Barcode Region and Primer Systems

We aimed to develop a DNA metabarcoding method allowing the differentiation between species belonging to the bivalve families Pectinidae, Ostreidae, and Mytilidae. To be applicable in routine analysis, the method should allow identifying the economically most important bivalve species in raw and highly processed food products.

We started with searching for appropriate DNA barcode regions of about 150 bp in length, containing conserved parts at the ends and a variable part in between. Potential DNA barcode regions were found in the mitochondrial DNA, especially the mitochondrial 16S rDNA. Several metabarcoding studies have shown that the sequences of the 16S rDNA gene are suitable as barcodes for species identification. Since we have already used a barcode region of the mitochondrial 16S rDNA to identify mammals and poultry [47], this marker gene was chosen as the DNA barcode for our assay.

**Figure 1:**
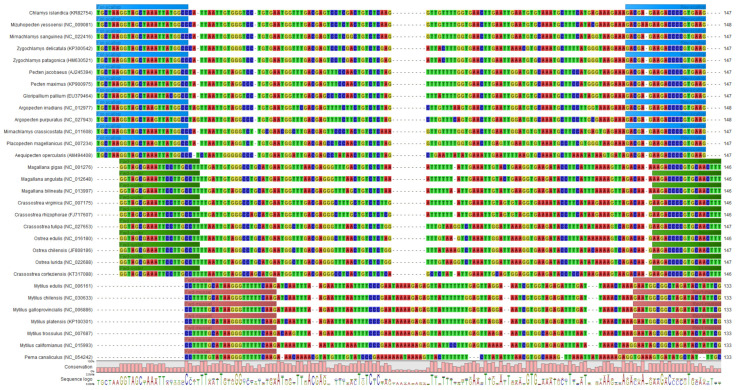
Multi-species sequence alignment of the mitochondrial 16S rDNA barcoding region for bivalve species. Colored bars indicate the binding sites of the primer sets for scallops (blue), oysters (green), and mussels (red, CLC Genomics Workbench software version 10.1.1, Qiagen, Hilden, Germany).

Since the DNA metabarcoding method for bivalves should be compatible with the DNA metabarcoding method for mammalian and poultry species published recently [47], the primers should anneal at the same temperature (62 °C). In addition, the PCR cycle number should be limited to 25 and DNA libraries should be sequenced with Illumina reagent kits in the 300-cycle format. Due to high sequence variability between closely related bivalve species, none of the primer sets designed enabled obtaining a PCR product for each of the bivalve species of interest. Thus, we continued by designing three primer sets, one for each of the three bivalve families, Pectinidae, Ostreidae, and Mytilidae. Primer pairs consisting of one forward and one reverse primer allowed amplifying the DNA barcode region in scallop and oyster species (Table 2). However, in the case of mussels, a primer set consisting of one forward primer and two reverse primers (Table 2) was necessary to obtain a PCR product for the mussel species listed in Table 1. Figure 1 shows an alignment of selected DNA barcode sequences for the commercially most relevant bivalve species. The alignment of the 90 bivalve species is shown in Supplementary Figure S1. Blue, green, and red bars indicate the binding sites of the primers for Pectinidae, Ostreidae and Mytilidae*,* respectively. With the three primer sets, PCR products differing in at least one base should be obtained for all bivalve species of interest.

Further sequence alignments indicated that the DNA barcode region selected does not allow distinguishing between all species of the following genera: *Chlamys* spp., *Euvola* spp., *Pecten* spp., *Crassostrea* spp., *Magallana* spp*., Ostrea* spp. and *Saccostrea* spp. These species cannot be distinguished: *Chlamys rubida* and *Chlamys behringiana*; *Pecten albicans*, *Pecten fumatus*, *Pecten jacobaeus*, *Pecten keppelianus*, *Pecten novaezelandiae*, *Pecten sulcicostatus*, *Crassostrea hongkongensis,* and *Crassostrea rivularis*; *Ostrea angelica* and *Ostrea lurida;* as well as *Ostrea permollis* and *Ostrea puelchana*; and *Saccostrea echinata*, *Saccostrea glomerata,* and *Saccostrea mytiloides*. In addition, two mussel species, *Mytilus platensis* and *Mytilus chilensis*, can also not be distinguished (for *Mytilus platensis* only one DNA sequence entry was in the public databases provided by NCBI). However, differentiation at the genus level (*Chlamys* spp., *Pecten* spp., *Crassostrea* spp., *Ostrea* spp., *Mytilus* spp.) is sufficient according to the “Codex Alimentarius Austriacus” chapter B35 [56].

When we tested the primers in singleplex PCR assays, for each of the reference samples a PCR product of about 150 bp in length was obtained by increasing the concentration of the forward primer for mussels to 0.4 µM and keeping the concentration of the other six primers at 0.2 µM. In addition, we tested whether the seven primers could be combined to a triplex system. PCR products for the bivalve species of interest were obtained in one and the same vial by increasing the MgCl_2_ concentration to a final concentration of 3 mM. Thus, we achieved our objective to perform the triplex PCR assay in combination with the previously published DNA metabarcoding assay for mammalian and poultry species [47].

### 3.2. Library Preparation, Pooling of Libraries, and Sequencing

Library preparation, pooling of 5 or 7 µL per normalized DNA library, and the sequencing process were performed as described previously [47]. However, in case of the pooling process, all DNA libraries were mixed in equal volumes as recommended by the manufacturer’s instruction. In our previous study, different volumes from individual DNA libraries were taken to achieve sufficient sequencing depth for minor components. For sample pooling to the maximum of 96 libraries, more than 100,000 NGS reads per sample were expected to be obtained using the 300-cycle MiSeq^®^ Reagent Kit v2.

Sequencing runs were performed in triplicate and the average run metrics were as follows: cluster density (969 K/mm^2^) on the flow cell, cluster passing filter (70.22%) as well as the Q-scores (Q30) for read 1 and read 2 were 92.6% and 89.28%, respectively. A total of 5.02% of the total reads were identified as PhiX control sequences with an error rate of 1.49%.

### 3.3. Analysis of DNA Extracts from Reference Samples

PCR products were obtained for each of the reference samples and sequencing results for those samples are summarized in Table 3. The table shows mean values of the total number of raw reads, the total number of reads that passed the analysis pipeline in Galaxy as well as the total number and percentage of reads that were assigned correctly to the eleven species (based on six replicates).

No significant differences were observed in the total number of reads (before data analysis process) between these species, except *Mytilus galloprovincialis* (162843), *Perna canaliculus* (169631*),* and *Mytilus edulis* (134500). With the exception of *Perna canaliculus*, >70% of the reads passed the amplicon analysis workflow. All three mussel species, six scallop species and two oyster species could be identified with this workflow at a high rate (>97.5%), except *Mytilus edulis*.

### 3.4. Analysis of DNA Extract Mixtures

Six ternary DNA extract mixtures were analyzed containing the DNA of the three bivalve families Pectinidae, Ostreidae, and Mytilidae in ratios of 98.0:1.5:0.5 (*v*/*v*/*v*). The composition of the DNA extract mixtures and the results obtained by DNA metabarcoding are summarized in Table 4. The total number of raw reads *ranged* from 80856 to 159,737 and the reads that passed the workflow were in the range from 65961 to 147196. For the main components (98.0%), the number of reads assigned correctly ranged from 62434 to 140147. In addition, both minor components (1.5% and 0.5%) could be identified. The number of reads assigned correctly was in the range from 1710 to 4356 and 555 to 1478, respectively.

In addition, we analyzed three DNA extract mixtures consisting of DNA from species belonging to one bivalve family (Table 5). The mixtures contained DNA from a scallop or mussel species, respectively. DNA from other bivalve species was present in a proportion of 1.0% each. Both species being present as main components, *Placopecten magellanicus* and *Perna canaliculus,* could be identified, with the number of reads assigned correctly ranging from 58156 to 77483. However, quite different numbers of reads were correctly assigned to the minor components, ranging from 626 (*Mizuhopecten yessoensis*) to 50,391 (*Mytilus galloprovincialis*). *Aequipecten opercularis* was the only minor component that could not be detected.

We analyzed a further DNA extract mixture containing DNA from the squid species *Sepiella inermis* as main component (97.0%) and DNA from the bivalve species *Placopecten magellanicus*, *Ostrea edulis,* and *Perna canaliculus* as minor components (1.0% each). As expected, in this mixture, the main component could not be detected because the primers are not suitable for amplification of the target region for *Sepiella inermis*. 31424, 28162, and 806 reads, respectively, were assigned correctly to the three bivalve species.

In our previous metabarcoding study [47], individual DNA libraries were pooled in different ratios to achieve sufficient sequencing depth for minor components. The present study demonstrates, that minor components down to a proportion of 0.5% could be identified and differentiated although DNA libraries were pooled by mixing them in equal volumes. DNA extracts from reference samples and DNA extract mixtures most frequently resulted in less than 100,000 reads. However, for all samples on average > 75000 raw reads were obtained, which turned out to be sufficient for reliable species identification.

### 3.5. Analysis of Commercial Seafood Samples

In order to investigate the applicability of the DNA metabarcoding method to foodstuffs, DNA extracts from 75 commercial food products were analyzed. According to declaration, eight samples (O1 and O4–O10) contained oyster species, 27 samples (M11, M14–M26, and M28–M40) mussel species, 15 samples (S41, S43–45, S48, S51–S55, and S56–S61) scallop species and 25 samples (Mi62–Mi86) were mixed-species seafood products (Table 6). The ingredient list of 30 out of 75 food products did not give any information on the bivalve species. A total of 39 samples were declared to contain “*Crassostrea gigas*”, “*Mytilus galloprovincialis”*, “*Mytilus chilensis”*, *“Mytilus edulis”*, *“Zygochlamys patagonica”*, *“Chlamys opercularis”*, *“Placopecten magellanicus”*, “*Pecten maximus*”, or *“Patinopecten yessoensis”*. The remaining samples (*n* = 6) were labelled with *“Mytilus* spp*.”* or *“Pecten* spp*.”*.

Our results indicate that DNA metabarcoding by targeting the 16S rDNA barcode region of about 150 bp in length is applicable to complex and highly processed foodstuffs. The barcode region could be amplified and sequenced even in products such as Bouillabaisse, Paella, and instant noodle seafood. Oyster sauce was the only sample matrix for which PCR amplification and consequently sequencing failed. Failure of obtaining PCR products for oyster sauce has already been reported by Chin Chin et al. [50], most probably caused by excessive DNA fragmentation due to industrial processing.

Three oyster species (*Saccostrea malabonensis*, *Magallana bilineata*, *Magallana gigas*), three mussel species (*Mytilus galloprovincialis*, *Mytilus edulis*, *Perna canaliculus*), and three scallop species (*Aequipecten opercularis*, *Placopecten magellanicus*, *Pecten* spp.) were detected in food products (O4, O8, M17, M19, M23, M25, M26, M28, M31, M32, M35, M38–M40, S51, S56, S58–S60, Mi63, Mi65, Mi70, Mi71, Mi73–Mi76, Mi81, Mi83, Mi85, and Mi86) although they were not declared on the label.

In each of the six oyster products that could be subjected to sequencing (O1, O4–O8), *Magallana gigas* was identified. *Magallana gigas* is by far the predominant oyster species farmed in the EU [59].

In 21 products (M11, M16, M18, M21, M24, M33–M35, M37, M39, M40, Mi62, Mi64, Mi66, Mi69, Mi72, Mi77–Mi80, and Mi84), the mussel species *Mytilus galloprovincialis* was detected. In addition to *Mytilus galloprovincialis*, *Mytilus edulis* was identified (percentage of reads assigned correctly >1%) in 13 products (M24, M33, M34, M39, Mi62, Mi64, Mi66, Mi69, Mi72, Mi78–Mi80, and Mi84). In four products, *Mytilus edulis* could not be detected although it was declared on the label. *Mytilus galloprovincialis* and *Mytilus edulis* are the two mussel species most frequently cultivated in European mussel farms [59]. In none of the products declared to contain *Mytilus chilensis, Mytilus chilensis* was detected. Instead of *Mytilus chilensis,* imported to EU countries from Chile [60], *Mytilus galloprovincialis* and/or *Mytilus edulis* were identified. According to the multi-species sequence alignment shown in Figure 1, the barcode region should allow distinguishing the three Mytilus species.

*Placopecten magellanicus* and *Patinopecten yessoensis* were listed as ingredients in samples S41, S45, S54, and S57 and samples S48, S52, and S61, respectively. Our results confirmed the presence of these two species, except for sample S57. In sample S43, declared to contain *Pecten maximus,* the species *Mizuhopecten yessoensis* was detected. In sample S44 and S53, declared as *Pecten* spp., the species *Mizuhopecten yessoensis* was also identified. In line with previous studies, most products declared to contain “Jakobsmuschel” did not contain a species of the genus *Pecten* [15,18,19]. Instead, we identified *Placopecten magellanicus* or *Mizuhopecten yessoensis*.

## 4. Conclusions

The DNA metabarcoding method developed in this study allows the detection of species of Mytilidae (mussels), Pectinidae (scallops), and Ostreidae (oysters), the most important bivalve families for human consumption. By combining three forward and four reverse primers in a triplex PCR assay, the barcode region, a fragment of mitochondrial 16S rDNA, could be amplified in the species of interest.

The applicability of the novel DNA metabarcoding method was investigated by analyzing individual DNA extracts from eleven reference samples, ten DNA extract mixtures and DNA extracts from 75 commercial food products. In each of the eleven reference samples, the bivalve species was identified correctly. In DNA extract mixtures, not only the main component but also the minor components were detected correctly, with just a few exceptions. The analysis of commercial seafood products showed that the DNA metabarcoding method is applicable to complex and processed foodstuffs, allowing the identification of bivalves in, e.g., marinated form, in sauces, in seafood mixes and even in instant noodle seafood.

The DNA metabarcoding method runs on both the MiSeq^®^ and iSeq^®^ instrument of Illumina. Due to the compatibility of PCR and sequencing parameters, the DNA metabarcoding method can be combined with a DNA metabarcoding method for mammalian and poultry species published recently.

## 5. Patent

This manuscript has been submitted for grant of a European patent (application number: EP21204456.4).

## Figures and Tables

**Table 1:** Bivalve species used for development of the DNA metabarcoding method.

Scientific Name	Commercial Name (German)	Commercial Name (English)
Mytilidae	Miesmuscheln	Mussels
*Mytilus edulis*	Gemeine Miesmuschel	Blue mussel
*Mytilus galloprovincialis*	Mittelmeer-Miesmuschel	Mediterranean mussel
*Perna canaliculus*	Neuseeland-Miesmuschel	New Zealand green-lipped mussel
Pectinidae	Kammmuscheln	Scallops
*Placopecten magellanicus*	Atlantischer Tiefseescallop	Atlantic deep-sea scallop
*Mizuhopecten yessoensis*	Japanische Kammmuschel	Yesso scallop
*Pecten jacobaeus*	Jakobsmuschel	Great scallop
*Zygochlamys patagonica*	Patagonische Kammmuschel	Patagonian scallop
*Argopecten purpuratus*	Purpur-Kammmuschel	Purple scallop
*Aequipecten opercularis*	Kleine Pilgermuschel	Queen scallop
Ostreidae	Austern	Oysters
*Magallana gigas*	Pazifische Felsenauster	Pacific oyster
*Ostrea edulis*	Europäische Auster	European flat oyster

**Table 2:** Primers designed in this study.

Name	Sequence 5′→3′
mussel	
For_Mu	CCTTTTGCATAAGGGTTTTTCAAG
Rev1_Mu	CGAATAGTATCTAGCCGCCATTC
Rev2_Mu	GCAAATAGCATATCACTTTCACCTC
scallop	
For_Mu	TGCTAAGGTAGCTAAATTATGGCC
Rev_Mu	CTTCACGGGGTCTTCTCGTC
oyster	
For_Mu	GGTAGCGAAATTCCTTGCCTT
Rev_Mu	AAAGTTGCACGGGGTCTT
overhang	
Forward	TCGTCGGCAGCGTCAGATGTGTATAAGAGACAG
Reverse	GTCTCGTGGGCTCGGAGATGTGTATAAGAGACAG

**Table 3:** Results for DNA extracts from reference samples. Numbers are mean values (*n* = 6, three sequencing runs, two replicates per run).

Sample ID	Declaration on the Product		Species Identified	Total Number of Raw Reads	Total Number	Number	Percentage
	Scientific/Latin Name	Product Description [Eng]			of Reads Passing the Workflow	of Reads Assigned Correctly	of Reads Assigned Correctly (%)
O2	*Ostrea edulis*	Oyster	*Ostrea edulis*	78559	63491	61875	97.46
O3	*Crassostrea gigas* *	Oyster	Magallana gigas *	76143	65389	64125	98.07
M12	*Mytilus* *galloprovincialis*	Blue Mussel	*Mytilus* *galloprovincialis*	162843	150678	149315	99.09
M13	*Perna canaliculus*	New Zealand green-lipped mussel	*Perna canaliculus*	169631	104861	103350	98.56
M27	*Mytilus edulis*	Mussels in marinade	*Mytilus edulis*	134500	120686	105024	87.02
S42	*Mizuhopecten yessoensis*	Yesso scallop	*Mizuhopecten yessoensis*	75927	58069	57058	98.26
S46	*Pecten jacobaeus*	Great scallop	*Pecten* spp.	79472	61484	60514	98.42
S47	*Zygochlamys patagonica*	Scallop “á la Bretonne”	*Zygochlamys* *patagonica*	77747	59245	58429	98.62
S49	*Placopecten magellanicus*	Great scallop	*Placopecten* *magellanicus*	79131	61531	60886	98.95
S50	*Argopecten purpuratus*	Pacific scallop	*Argopecten* *purpuratus*	77383	55455	54588	98.44
S55	*Aequipecten opercularis*	Scallop in sauce	*Aequipecten* *opercularis*	79141	56064	55800	99.53

* former nomenclature, synonym for *Magallana gigas*.

**Table 4:** Results for ternary DNA extract mixtures representing the three bivalve families of interest. DNA extracts (5 ng/µL) were mixed in a ratio of 98.0:1.5:0.5 (*v*/*v*/*v*). Numbers are mean values (*n* = 9, three sequencing runs, three replicates per run).

	Proportion		Total Number of Raw Reads	Total Number of Reads Passing the Workflow	Reads Assigned Correctly					
Species 1 (98%)	Species 2 (1.5%)	Species 3 (0.5%)			Species 1	(%)	Species 2	(%)	Species 3	(%)
*Magallana* *gigas*	*Mytilus* *galloprovincialis*	*Pecten* spp.	80856	69506	66430	95.57	1985	2.86	658	0.95
*Magallana* *gigas*	*Pecten* spp.	*Mytilus* *galloprovincialis*	89552	76669	73114	95.36	2182	2.85	894	1.17
*Pecten* spp.	*Magallana* *gigas*	*Mytilus* *galloprovincialis*	88971	69682	66291	95.13	1710	2.45	922	1.32
*Pecten* spp.	*Mytilus* *galloprovincialis*	*Magallana* *gigas*	84085	65961	62434	94.65	2281	3.46	555	0.84
*Mytilus* *galloprovincialis*	*Pecten* spp.	*Magallana* *gigas*	159737	147196	140147	95.21	4356	2.96	1478	1.00
*Mytilus* *galloprovincialis*	*Magallana* *gigas*	*Pecten* spp.	147443	136629	130986	95.87	3304	2.42	1156	0.85

**Table 5:** Results for DNA extract mixtures representing one bivalve family. DNA from minor components was present in a proportion of 1% each. In addition, results for a DNA extract mixture containing DNA from a squid species (*Sepiella inermis*) as main component (97.0%) and DNA from three bivalve species (1% each) is shown. Numbers are mean values (*n* = 9, three sequencing runs, three replicates per run).

Main Component	Minor Component (1.0% Each)	Total Number of Raw Reads	Total Number of Reads Passed the Workflow	Reads Assigned Correctly	Percentage of Reads Assigned Correctly (%)
*Placopecten magellanicus*		83526 *	65446	58156	88.86
	*Mizuhopecten yessoensis*			626	0.96
	*Pecten* spp.			817	1.25
	*Zygochlamys patagonica*			4534	6.93
	*Argopecten purpuratus*			663	1.01
	*Aequipecten opercularis*			35	0.05
*Placopecten magellanicus*		84282 *	66691	63628	95.41
	*Magallana gigas*			1298	1.95
	*Ostrea edulis*			1088	1.63
*Perna canaliculus*		179227 *	128882	77483	60.12
	*Mytilus galloprovincialis*			50391	39.10
	*Mytilus edulis*			824	0.64
*Sepiella inermis*		78467	61415		
	*Placopecten magellanicus*			31424	51.17
	*Ostrea edulis*			28162	45.86
	*Perna canaliculus*			806	1.31

* Number of values (*n* = 6, three sequencing runs, two replicates per run).

**Table 6:** Results obtained for commercial seafood samples. Samples listed above the double line were sequenced with the MiSeq^®^ (three sequencing runs, one replicate per run, numbers are mean values); samples listed below the double line were sequenced either with the MiSeq^®^ or the iSeq^®^.

Sample ID	Declaration on the Product		Species Identified	Total Number of Raw Reads	Total Number of Reads Passed the Workflow	Reads Assigned Correctly	Percentage of Reads Assigned Correctly (%)
	Scientific/Latin Name	Product Description [Eng]					
O5	*Crassostrea gigas* ^4^	Oyster in sunflower oil	*Magallana gigas* ^4^	76930 ^1^	65728	64369	97.93
O6	*Crassostrea gigas* ^4^	Oyster in sunflower oil	*Magallana gigas* ^4^	44848 ^1^	38547	37610	97.57
O7	*Crassostrea gigas* ^4^	Oyster in water	*Magallana gigas* ^4^	76247	64917	63700	98.13
O8	not declared	Oyster sauce	*Saccostrea malabonensis*	14470	11658	5442	46.68
			*Magallana bilineata*			4652	39.91
M23	not declared	Mussel with sherry vinegar	*Mytilus galloprovincialis*	33517	30794	30358	98.58
M25	not declared	Mussel in marinade sauce	*Mytilus galloprovincialis*	163188	151688	150700	99.35
M26	not declared	Grilled blue mussel	*Mytilus galloprovincialis*	163106	151608	150433	99.23
M29	*Mytilus galloprovincialis*	Blue mussel in	*Mytilus galloprovincialis*	153435	140475	132354	94.22
		tomato sauce	*Mytilus edulis*			7937	5.65
M30	*Mytilus galloprovincialis*	Blue mussel	*Mytilus galloprovincialis*	185479	171890	170624	99.26
		A la mariniere	*Mytilus edulis*			1156	0.67
M31	not declared	Blue mussel in	*Mytilus galloprovincialis*	170303	158379	157015	99.14
		organic marinade	*Mytilus edulis*			1267	0.80
M32	not declared	Marinated blue	*Mytilus galloprovincialis*	159181	144788	143399	99.04
		mussel	*Mytilus edulis*			1308	0.90
M33	*Mytilus chilensis*	Mussel in	*Mytilus galloprovincialis*	167903	151219	118879	78.61
		Escabeche	*Mytilus edulis*			31737	20.99
M34	*Mytilus chilensis*	Mussel	*Mytilus galloprovincialis*	152112	138768	87964	63.39
			*Mytilus edulis*			49601	35.74
M36	*Mytilus galloprovincialis*	Blue mussel marinated	*Mytilus galloprovincialis* *Mytilus edulis*	176963	163721	162224	99.09
						1323	0.81
M37	*Mytilus edulis*	Mussel in honey mustard sauce	*Mytilus galloprovincialis* *Mytilus edulis*	149364	136868	135249	98.82
						1400	1.02
M38	not declared	Blue mussel	*Mytilus galloprovincialis*	138801	127244	125980	99.01
		in marinade	*Mytilus edulis*			1056	0.83
S58	not declared	Rillettes de	*Aequipecten opercularis*	62787	44307	42716	96.41
		Saint-Jacques	*Mytilus galloprovincialis*			1330	3.00
S59	not declared	Small scallop in galician sauce	*Aequipecten opercularis*	82550	59722	58296	97.61
Mi62	*Mytilus chilensis*	Seafood mix	*Mytilus galloprovincialis*	618324	569815	433439	76.07
			*Mytilus edulis*			134543	23.61
Mi63	not declared	Sauce with	*Mytilus edulis*	152170	139306	73550	52.80
		seafood	*Mytilus galloprovincialis*			64729	46.47
Mi64	*Mytilus chilensis* *Mytilus edulis*	Seafood mix	*Mytilus galloprovincialis* *Mytilus edulis*	131285	119350	81590	68.36
						37211	31.18
Mi65	not declared	Bouillabaisse Marseille	*Mytilus galloprovincialisMytilus edulis*	157311	143479	138535	96.55
						4777	3.33
Mi66	*Mytilus chilensis*	Seafood mix	*Mytilus galloprovincialis*	152535	140047	92024	65.71
			*Mytilus edulis*			47415	33.86
Mi67	*Mytilus* spp.	Seafood mix	*Mytilus galloprovincialis*	76544	69081	48275	69.88
			*Mytilus edulis*			20459	29.62
Mi68	*Mytilus galloprovincialis*	Sea fruit salad in sunflower oil	*Mytilus galloprovincialis* *Mytilus edulis*	157861	145671	144468	99.17
						1046	0.72
Mi69	*Mytilus chilensis*	Seafood mix	*Mytilus galloprovincialis* *Mytilus edulis*	140227	128007	85679	66.93
						41686	32.57
Mi70	not declared	*Sea fruit salad* *fantasy*	*Mytilus galloprovincialis* *Mytilus edulis*	120677	106674	101121	94.80
						5413	5.07
Mi71	not declared	Seafood mix	*Mytilus galloprovincialis*	160546	147278	79680	54.10
			*Mytilus edulis*			66675	45.27
Mi72	*Mytilus chilensis*	Seafood mix	*Mytilus galloprovincialis*	160059	146539	91557	62.48
			*Mytilus edulis*			54271	37.03
Mi73	not declared	Seafood mix	*Mytilus edulis*	150500	137634	78942	57.36
			*Mytilus galloprovincialis*			57608	41.86
Mi74	not declared	Seafood mix	*Mytilus galloprovincialis*	168841	155701	79035	50.76
			*Mytilus edulis*			75612	48.56
Mi75	not declared	Pizza Frutti di	*Mytilus galloprovincialis*	181822 ^1^	172620	95184	55.14
		mare	*Mytilus edulis*			71440	41.39
Mi76	not declared	Paella	*Mytilus galloprovincialis*	150431	139511	138335	99.16
			*Mytilus edulis*			1070	0.77
Mi77	*Mytilus edulis,*	Paella	*Mytilus galloprovincialis*	141816	132092	130768	99.00
	*Mytilus chilensis*		*Mytilus edulis*			1242	0.94
Mi78	*Mytilus chilensis*	Seafood all’Olio	*Mytilus galloprovincialis*	134717	122906	73482	59.79
			*Mytilus edulis*			48774	39.68
Mi79	*Mytilus chilensis*	Seafood mix	*Mytilus galloprovincialis*	148773	137122	73035	53.26
			*Mytilus edulis*			63249	46.13
Mi80	*Mytilus chilensis*	Seafood mix	*Mytilus galloprovincialis*	136695	126608	88130	69.61
			*Mytilus edulis*			37970	29.99
Mi81	not declared	Sea fruit salad	*Mytilus galloprovincialis*	153499	142736	141578	99.19
			*Mytilus edulis*			1022	0.72
Mi82	*Zygochlamys patagonica* *Chlamys opercularis*	Scallop terrine	*Zygochlamys patagonica*	76554	59181	57329	96.87
Mi83	not declared	Terrine of salmon and great scallop	*Pecten* spp.	96596 ^1^	76834	75476	98,23
Mi84	*Mytilus chilensis*	Seafood mix	*Mytilus galloprovincialis*	163885	150852	124468	82.51
			*Mytilus edulis*			25916	17.18
Mi85	not declared	Instant noodle seafood, mild	*Mytilus galloprovincialis*	15409	14118	13750	97.39
Mi86	not declared	Instant noodle seafood, spicy	*Mytilus galloprovincialis*	9787	8892	8473	95.29
O1	*Crassostrea gigas* ^4^	Oyster	*Magallana gigas* ^4^	139319 ^2^	134073	133493	99.57
O4	not declared	Oyster	*Magallana gigas*	46089 ^2^	40991	40279	98.26
O9	not declared	Oyster sauce		not evaluable ^3^			
O10	not declared	Oyster sauce		not evaluable ^3^			
M11	*Mytilus edulis*	Mussel	*Mytilus galloprovincialis*	23766 ^2^	22546	22147	98.23
M14	*Mytilus* spp.	Blue mussel	*Mytilus galloprovincialis*	126880 ^2^	119717	79522	66.42
			*Mytilus edulis*			39555	33.04
M15	*Mytilus* spp	Blue mussel	*Mytilus galloprovincialis*	227678	220699	220226	99.79
M16	*Mytilus edulis*	Bouchot mussel	*Mytilus galloprovincialis*	51292 ^2^	49604	48832	98.44
M17	not declared	Grilled blue	*Mytilus galloprovincialis*	9888 ^2^	6750	3956	58.61
		mussel	*Mytilus edulis*			1998	29.60
M18	*Mytilus chilensis*	Blue mussel	*Mytilus galloprovincialis*	53710 ^2^	51670	50733	98.19
M19	not declared	Blue mussel	*Mytilus galloprovincialis*	57238 ^2^	54822	53829	98.19
M20	*Mytilus* spp.	Blue mussel	*Mytilus galloprovincialis*	72113 ^2^	69576	68969	99.13
M21	*Mytilus edulis*	Mussel	*Mytilus galloprovincialis*	51328 ^2^	49908	49459	99.10
M22	*Mytilus galloprovincialis*	Blue mussel	*Mytilus galloprovincialis*	115950^2^	110777	109262	98.63
			*Mytilus edulis*			1466	1.32
M24	*Mytilus chilensis*	Blue mussel in	*Mytilus galloprovincialis*	113942 ^2^	107150	94449	88.15
		tomato sauce	*Mytilus edulis*			12505	11.67
M28	not declared	Dry cat food	*Pecten* spp.	128693 ^3^	126380	79764	63.11
		with green	*Mytilus galloprovincialis*			40450	32.01
		lipped mussel	*Perna canaliculus*			4712	3.73
M35	*Mytilus chilensis*	Mussel in tomato sauce	*Mytilus galloprovincialis*	197899 ^3^	190771	189540	99.35
M39	*Mytilus chilensis*	Blue mussel	*Mytilus galloprovincialis*	182612 ^3^	175982	96502	54.84
			*Mytilus edulis*			75204	42.73
M40	*Mytilus edulis*	Blue mussel	*Mytilus galloprovincialis*	182958 ^3^	179399	178024	99.23
S41	*Placopecten magellanicus*	Deep-sea scallop	*Placopecten magellanicus*	143794 ^2^	132140	131583	99.58
S43	*Pecten maximus*	Great scallop	*Mizuhopecten yessoensis*	122156 ^2^	113706	113128	99.49
S44	*Pecten* spp.	Great scallop	*Mizuhopecten yessoensis*	2873135 ^2^	2718126	2717426	99.97
S45	*Placopecten magellanicus*	Deep-sea scallop	*Placopecten magellanicus*	111673 ^2^	107119	106632	99.55
S48	*Patinopecten yessoensis*	Great scallop/ Yesso scallop	*Mizuhopecten yessoensis*	47397 ^2^	41076	407873	99.51
S51	not declared	Great scallop	*Placopecten magellanicus*	51565 ^2^	45007	44915	99.80
S52	*Patinopecten yessoensis*	Great scallop	*Mizuhopecten yessoensis*	46673 ^2^	39769	39627	99.64
S53	*Pecten* spp.	Great scallop	*Mizuhopecten yessoensis*	42857 ^2^	36443	35265	96.77
S54	*Placopecten magellanicus*	Great scallop	*Placopecten magellanicus*	55475 ^2^	48703	47915	98.38
S56	not declared	Great scallop	*Placopecten magellanicus*	1268169 ^3^	1061137	1060653	99.95
S57	*Placopecten magellanicus*	Great scallop	*Pecten* spp.	174497 ^3^	171299	170404	99.48
S60	not declared	Deep-sea scallop	*Placopecten magellanicus*	364474 ^3^	350953	350869	99.98
S61	*Patinopecten yessoensis*	Great scallop	*Mizuhopecten yessoensis*	159145 ^3^	152930	152849	99.95

^1^ Mean of two replicates; ^2^ samples were analyzed with the MiSeq^®^ instrument; ^3^ samples were analyzed with the iSeq^®^ instrument; ^4^ former nomenclature, synonym for *Magallana gigas*.

## Data Availability

The datasets generated for this study are available on request to the corresponding author.

## Supplementary Materials

The following are available online at https://www.mdpi.com/article/10.3390/foods10112618/s1, Supplementary Table S1: Declaration, origin and processing condition of the 86 food products, Supplementary Table S2: Sequences included into the reverence database, Supplementary Figure S1: Multi-species sequence alignment of the mitochondrial 16S rDNA barcoding region for the bivalve species of interest.
