# Supplementary material for: Development of a DNA Metabarcoding Method for the Identification of Bivalve Species in Seafood Products
Source: Foods. 2021 Oct 28;10(11):2618. doi: 10.3390/foods10112618 (PMC8617786; doi:10.3390/foods10112618)
Supplement: Supplementary file 1 [file foods-10-02618-s001.zip › Supplementary_MOS_4.pdf]

Appendix A

Supplementary Table S1 Declaration, origin and processing condition of the 86 commercial food products.

	Declaration on the product			*		
**Sample ID**	**Scientific/Latin name**	**Product description [Ger]**	**Product description [Eng]**	**Product origin**	**Purchase origin**	**Treatment**
O1	*Crassostrea gigas*	Felsenauster	Giant Oyster	France	delicacy shops	raw
O2	*Ostrea edulis*	Auster	Oyster	Denmark	fish market	raw
O3	*Crassostrea gigas*	Auster / Gillardeau	Oyster / Gillardeau	Denmark	fish market	raw
O4	not declared	Auster	Oyster	Pacific *	delicacy shops	raw
O5	*Crassostrea gigas*	Auster in Sonnenblumenöl	Oyster in sunflower oil	Korea	delicacy shops	processed
O6	*Crassostrea gigas*	Auster in Sonnenblumenöl	Oyster in sunflower oil	Korea	regional supermarket	processed
O7	*Crassostrea gigas*	Auster in Wasser	Oyster in water	Korea	delicacy shops	processed
O8	not declared	Austernsauce	Oyster sauce	Thailand	regional supermarket	processed
O9	not declared	Austernsauce	Oyster sauce	Thailand	delicacy shops	processed
O10	not declared	Austernsauce	Oyster sauce	China	delicacy shops	processed
M11	*Mytilus edulis*	Pfahlmuschel	Mussel	Spain	delicacy shops	processed
M12	*Mytilus galloprovincialis*	Miesmuschel	Blue Mussel	Spain	regional supermarket	frozen
M13	*Perna canaliculus*	Grünlippmuschel	New Zealand green-lipped mussel	New Zealand	delicacy shops	frozen
M14	*Mytilus spp.*	Miesmuschel	Blue Mussel	not declared	not declared	frozen
M15	*Mytilus edulis*	Miesmuschel	Blue Mussel	North Atlantic *	delicacy shops	raw
M16	*Mytilus edulis*	Bouchotmuschel	Bouchot mussel	France	fish market	raw
M17	not declared	Gegrillte Miesmuschel	Grilled blue mussel	Italy	delicacy shops	processed
M18	*Mytilus chilensis*	Miesmuschel	Blue Mussel	Spain	delicacy shops	frozen
M19	not declared	Miesmuschel	Blue Mussel	not declared	fish market	raw
M20	*Mytilus spp.*	Miesmuschel	Blue Mussel	not declared	fish market	raw
M21	*Mytilus edulis*	Muschel	Mussel	Denmark	delicacy shops	processed
M22	*Mytilus galloprovincialis*	Miesmuschel	Blue Mussel	Italy	delicacy shops	processed
M23	not declared	Muschel mit Sherry Essig	Mussel with sherry vinegar	Spain	delicacy shops	processed
M24	*Mytilus chilensis*	Miesmuschel in Tomatensauce	Blue Mussel in tomato sauce	Denmark	regional supermarket	processed
M25	not declared	Pfahlmuschel in Marinaden Sauce	Mussel in marinade sauce	Spain	regional supermarket	processed
M26	not declared	Gegrillte Miesmuschel	Grilled blue mussel	Italy	regional supermarket	processed
M27	*Mytilus chilensis*	Muschel in Marinade	Mussel in marinade	Spain	regional supermarket	processed
M28	not declared	Katzentrockenfutter mit Grünlippmuschel	Dry cat food with green lipped mussel	Germany	delicacy shops	processed
M29	*Mytilus galloprovincialis*	Miesmuschel in Tomatensauce	Blue mussel in tomato sauce	Spain	regional supermarket	processed

M30	*Mytilus galloprovincialis*	Miesmuschel a la mariniere	Blue mussel a la mariniere	Spain	regional supermarket	processed
M31	not declared	Miesmuschel in Bio-Marinade	Blue mussel in organic marinade	Germany	delicacy shops	processed
M32	not declared	Marinierte Miesmuschel	Marinated blue mussels	Spain	delicacy shops	processed
M33	*Mytilus chilensis*	Muschel in Escabeche	Mussel in Escabeche	Denmark	delicacy shops	processed
M34	*Mytilus chilensis*	Muschel	Mussel	Denmark	delicacy shops	processed
M35	*Mytilus chilensis*	Muschel in Tomatensauce	Mussel in tomato sauce	Denmark	delicacy shops	processed
M36	*Mytilus galloprovincialis*	Miesmuschel mariniert	Blue mussel marinated	Germany	delicacy shops	processed
M37	*Mytilus edulis*	Muschel in Honigsenfsauce	Mussel in honey mustard sauce	Denmark	delicacy shops	processed
M38	not declared	Miesmuschel in Marinade	Blue mussel in marinade	Spain	delicacy shops	processed
M39	*Mytilus chilensis*	Miesmuschel	Blue mussel	Germany	not declared	processed
M40	*Mytilus edulis*	Miesmuschel	Blue mussel	Netherlands	not declared	raw
S41	*Placopecten magellanicus*	Tiefseescallop	Deep-sea scallop	France	regional supermarket	cooled
S42	*Mizuhopecten yessoensis*	Japanische Kammmuschel	Yesso scallop	North Atlantic	fish market	cooled
S43	*Pecten maximus*	Jakobsmuschel	Great scallop	Pacific *	delicacy shops	cooled
S44	*Pecten spp.*	Jakobsmuschel	Great scallop	not declared	not declared	frozen
S45	*Placopecten magellanicus*	Tiefseescallop	Deep-sea scallop	not declared	not declared	frozen
S46	*Pecten jacobaeus*	Jakobsmuschel	Great scallop	Croatia	delicacy shops	frozen
S47	*Zygochlamys patagonica*	Jakobsmuschel "á la Bretonne"	Scallop "á la Bretonne"	France	delicacy shops	processed
S48	*Patinopecten yessoensis*	Jakobsmuschel/ Japanische Kammmuschel	Great scallop/ Yesso scallop	Pacific *	fish market	cooled
S49	*Placopecten magellanicus*	Jakobsmuschel	Great scallop	Pacific *	delicacy shops	cooled
S50	*Argopecten purpuratus*	Purpur Kammmuschel	Pacific scallop	Peru	delicacy shops	frozen
S51	not declared	Jakobsmuschel	Great scallop	not declared	fish market	cooled
S52	*Patinopecten yessoensis*	Jakobsmuschel	Great scallop	Pacific *	delicacy shops	cooled
S53	*Pecten sp.*	Jakobsmuschel	Great scallop	not declared	fish market	cooled
S54	*Placopecten magellanicus*	Jakobsmuschel	Great scallop	Pacific *	delicacy shops	cooled
S55	*Aequipecten opercularis*	Kammmuschel in Sauce	Scallop in sauce	Spain	delicacy shops	processed
S56	not declared	Jakobsmuschel	Great scallop	not declared	restaurant	processed
S57	*Placopecten magellanicus*	Jakobsmuschel	Great scallop	Austria	fish market	cooled
S58	not declared	Rillettes de Saint-Jacques	Rillettes de Saint-Jacques	France	delicacy shops	processed
S59	not declared	Kleine Pilgermuschel in galizischer Sauce	Small scallop in galician sauce	Spain	delicacy shops	processed

S60	not declared	Tiefseescallop	Deep-sea scallop	Germany	not declared	frozen
S61	*Patinopecten yessoensis*	Jakobsmuschel	Great scallop	Pacific *	delicacy shops	cooled
Mi62	*Mytilus chilensis*	Meeresfürchte Mischung	Seafood mix	France	regional supermarket	frozen
Mi63	not declared	Sauce mit Meeresfrüchten	Sauce with seafood	Italy	regional supermarket	processed
Mi64	*Mytilus chilensis, Mytilus edulis*	Meeresfrüchte Mischung	Seafood mix	Germany	delicacy shops	processed
Mi65	not declared	Bouillabaise Marseiller Art	Bouillabaise Marsille	Germany	delicacy shops	processed
Mi66	*Mytilus chilensis*	Meeresfrüchte Mischung	Seafood mix	France	regional supermarket	processed
Mi67	*Mytilus spp.*	Meeresfrüchte Mischung	Seafood mix	Chile	regional supermarket	processed
Mi68	*Mytilus galloprovincialis*	Meeresfrüchtesalat in Sonnenblumenöl	Sea fruit salad in sunflower oil	Italy	regional supermarket	processed
Mi69	*Mytilus chilensis*	Meeresfrüchte Mischung	Seafood mix	France	delicacy shops	processed
Mi70	not declared	Meeresfrüchtesalat Fantasie	Sea fruit salad fantasy	Italy	regional supermarket	processed
Mi71	not declared	Meeresfrüchte Mix	Seafood mix	Italy	regional supermarket	processed
Mi72	*Mytilus chilensis*	Meeresfrüchte Mix	Seafood mix	Croatia	delicacy shops	processed
Mi73	not declared	Meeresfrüchte Mix	Seafood mix	not declared	not declared	unknown
Mi74	not declared	Meeresfrüchte Mix	Seafood mix	not declared	not declared	unknown
Mi75	not declared	Pizza Frutti di Mare	Pizza Frutti di mare	Austria	restaurant	processed
Mi76	not declared	Paella	Paella	Germany	delicacy shops	processed
Mi77	*Mytilus edulis, Mytilus chilensis*	Paella	Paella	Germany	regional supermarket	processed
Mi78	*Mytilus chilensis*	Meeresfüchte all´Olio	Seafood all´Olio	France	regional supermarket	processed
Mi79	*Mytilus chilensis*	Meeresfrüchte Mix	Seafood mix	Spain	delicacy shops	processed
Mi80	*Mytilus chilensis*	Meeresfrüchte Mix	Seafood mix	Austria	delicacy shops	processed
Mi81	not declared	Meeresfrüchtesalat	Sea fruit salad	Italy	delicacy shops	processed
Mi82	*Zygochlamys patagonica, Chlamys opercularis*	Jakobsmuschelterrine	Scallop terrine	France	delicacy shops	processed
Mi83	not declared	Terrine vom Lachs und Jakobsmuschel	Terrine of salmon and great scallop	Austria	delicacy shops	processed
Mi84	*Mytilus chilensis*	Meeresfrüchte Mischung	Seafood mix	Germany	delicacy shops	processed
Mi85	not declared	Instant Nudeln Seafood, mild	Instant noodle seafood, mild	Korea	delicacy shops	processed
Mi86	not declared	Instant Nudeln Seafood, scharf	Instant noodle seafood, spicy	Korea	delicacy shops	processed

* In case the country of production was unknown, the fishing region was specified

Supplementary Table S2 Sequences included into the reference database.

Scientific name of species	Accession No	Scientific name of species	Accession No
**mussels**		**scallops**	
Mytilus chilensis	NC_030633	*Euvola vogdesi*	AJ972431
Perna perna	NC_026288	*Mimachlamys crassicostata*	NC_011608
Mytilus unguiculatus	NC_024733	*Gloripallium pallium*	EU379464
Perna viridis	NC_018362	*Flexopecten glaber*	AJ243574
Mytilus californianus	NC_015993	*Pecten jacobaeus*	AJ245394
Mytilus trossulus	NC_007687	*Pecten novaezelandiae*	AY650055
Mytilus galloprovincialis	NC_006886	*Euvola raveneli*	EU379473
Mytilus edulis	NC_006161	*Aequipecten opercularis*	AM494408
Perna canaliculus	NC_054242	*Euvola perula*	HM630517
Mytilus platensis	KP100301	*Nodipecten nodosus*	GQ342275
		Scaeochlamys livida	GQ166559
**oysters**		*Pecten keppelianus*	FN667667
Magallana bilineata	NC_013997	*Talochlamys multistriata*	FN667665
Magallana gigas	NC_001276	*Patinopecten caurinus*	FJ263642
Crassostrea virginica	NC_007175	*Chlamys behringiana*	FJ263641
Magallana hongkongensis	NC_011518	*Placopecten septemradiatus*	EU379475
Magallana angulata	NC_012648	*Pecten maximus*	KP900975
Magallana sikamea	NC_012649	*Pecten albicans*	KP900974
Magallana ariakensis	NC_012650	*Zygochlamys delicatula*	KP300542
Ostrea denselamellosa	NC_015231	*Chlamys hastata*	KF982789
Magallana nippona	NC_015248	*Ylistrum japonicum*	KF982785
Ostrea edulis	NC_016180	*Pecten fumatus*	JF339109
Ostrea lurida	NC_022688	*Talochlamys gemmulata*	JF339106
Crassostrea tulipa	NC_027653	*Zygochlamys patagonica*	HM630521
Ostrea angasi	AF052063	*Argopecten purpuratus*	NC_027943
Magallana belcheri	NC_037851	*Argopecten irradians*	NC_012977
Crassostrea rhizophorae	FJ717607	*Azumapecten farreri*	NC_012138
Crassostrea brasiliana	HQ711627	*Mizuhopecten yessoensi*	NC_009081
Talonostrea talonata	KX364275	*Placopecten magellanicus*	NC_007234
Crassostrea corteziensis	KT317088	*Euvola ziczac*	EU379485
Magallana rivularis	AY510450	*Pecten sulcicostatus*	KU754458
Ostrea angelica	KT317130	*Chlamys islandica*	KR827548
Ostrea permollis	AF052075	*Argopecten ventricosus*	KT161261
Ostrea chilensis	JF808186	*Mimachlamys varia*	KT988340
Ostrea algoensis	AF052062	*Amusium pleuronectes*	KP900978
Ostrea puelchana	AF052073	*Mimachlamys sanguinea*	NC_022416
Ostrea megodon	KX364274	*Talochlamys dichroa*	KP300543
Saccostrea cuccullata	NC_027724	*Mimachlamys gloriosa*	KP300549
Saccostrea palmula	KT317278	*Mimachlamys cloacata*	JF339118
Saccostrea malabonensis	KX961677	*Mimachlamys asperrima*	JF339117
Saccostrea scyphophilla	NC_013998	*Annachlamys striatula*	KF982786

Saccostrea glomerata	NC_036483	*Decatopecten radula*	KF982788
Saccostrea kegaki	NC_030533	*Bractechlamys vexillum*	KF982787
Saccostrea echinata	NC_036478	*Aequipecten glyptus*	EU379445
Saccostrea mytiloides	NC_036479	*Scaeochlamys lemniscata*	KP300554
		Chlamys rubida	FJ263645
		Karnekampia sulcata	JF496755
		Crassadoma gigantea	FJ263644
		Ylistrum balloti	JF339127

Supplementary Figure S1 Multi-species sequence alignment of the mitochondrial 16S rDNA barcoding region for the bivalve species of interest. Colored bars indicate the binding sites of the primer sets for scallops (blue), oysters (green) and mussels (red, CLC Genomics Workbench 10.1.1 (Qiagen)).

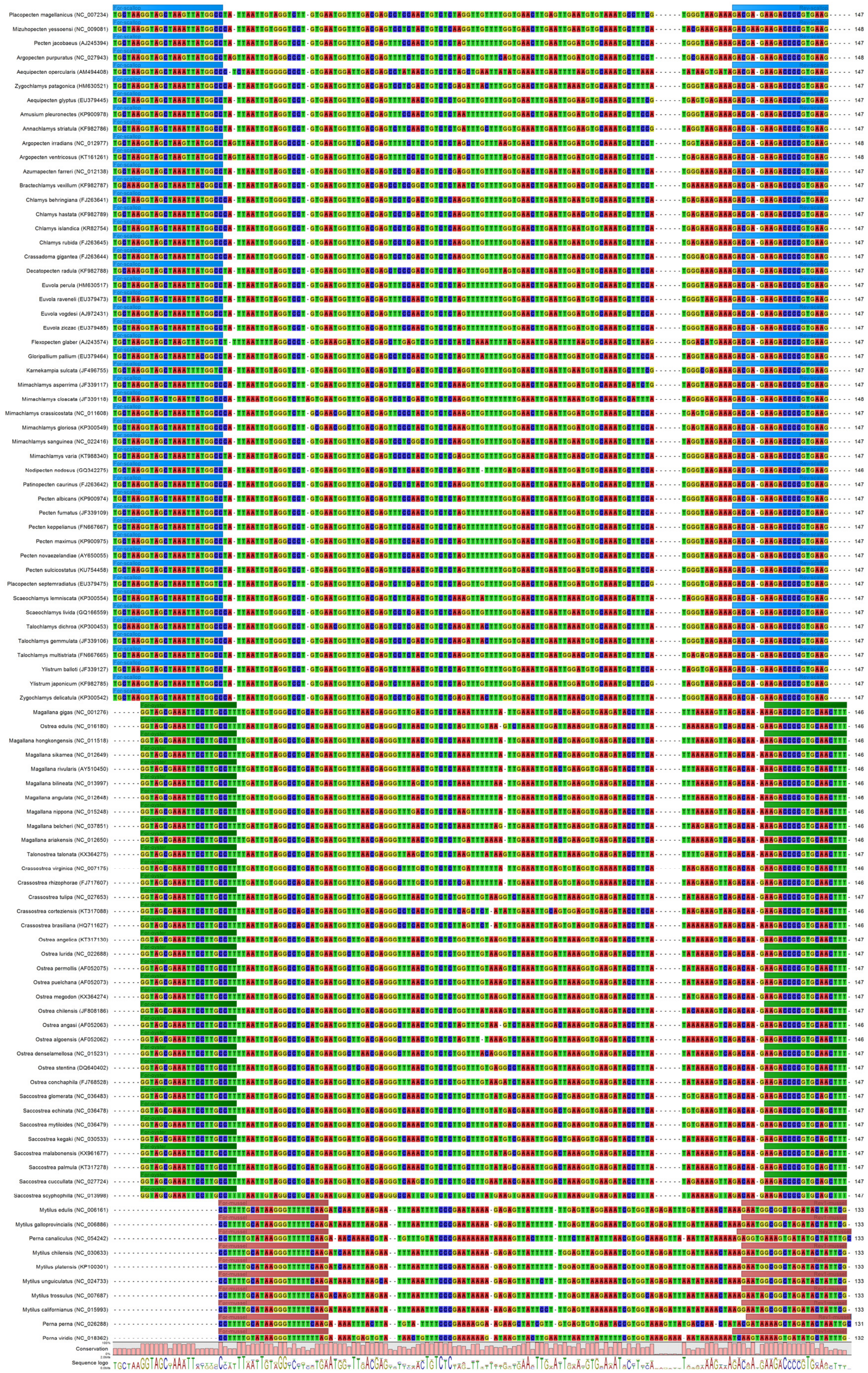
For-scallop
Placopecten magellanicus (NC_007234)
Mizuhopecten yessoensi (NC_009081)
Pecten jacobaeus (AJ245394)
Argopecten purpuratus (NC_027943)
Aequipecten opercularis (AM494408)
Zygochlamys patagonica (HM630521)
Aequipecten glyptus (EU379445)
Amusium pleuronectes (KP900978)
Annachlamys striatula (KF982786)
Argopecten irradians (NC_012977)
Argopecten ventricosus (KT161261)
Azumapecten farreri (NC_012138)
Bractechlamys vexillum (KF982787)
Chlamys behringiana (FJ263641)
Chlamys hastata (KF982789)
Chlamys islandica (KR82754)
Chlamys rubida (FJ263645)
Crassadoma gigantea (FJ263644)
Decatopecten radula (KF982788)
Euvola perula (HM630517)
Euvola raveneli (EU379473)
Euvola vogdesi (AJ972431)
Euvola ziczac (EU379485)
Flexopecten glaber (AJ243574)
Gloripallium pallium (EU379464)
Karnekampia sulcata (JF496755)
Mimachlamys asperrima (JF339117)
Mimachlamys cloacata (JF339118)
Mimachlamys crassicostata (NC_011608)
Mimachlamys gloriosa (KP300549)
Mimachlamys sanguinea (NC_022416)
Mimachlamys varia (KT988340)
Nodipecten nodosus (GQ342275)
Patinopecten caurinus (FJ263642)
Pecten albicans (KP900974)
Pecten fumatus (JF339109)
Pecten keppelianus (FN667667)
Pecten maximus (KP900975)
Pecten novaezelandiae (AY650055)
Pecten sulcicostatus (KU754458)
Placopecten septemradiatus (EU379475)
Scaeochlamys lemniscata (KP300554)
Scaeochlamys livida (GQ166559)
Talochlamys dichroa (KP300453)
Talochlamys gemmulata (JF339106)
Talochlamys multistriata (FN667665)
Ylistrum balloti (JF339127)
Ylistrum japonicum (KF982785)
Zygochlamys delicatula (KP300542)
Magallana gigas (NC_001276)
Ostrea edulis (NC_016180)
Magallana hongkongensis (NC_011518)
Magallana sikamea (NC_012649)
Magallana rivularis (AY510450)
Magallana bilineata (NC_013997)
Magallana angulata (NC_012648)
Magallana nippona (NC_015248)
Magallana belcheri (NC_037851)
Magallana ariakensis (NC_012650)
Talonostrea talonata (KX364275)
Crassostrea virginica (NC_007175)
Crassostrea rhizophorae (FJ717607)
Crassostrea tulipa (NC_027653)
Crassostrea corteziensis (KT317088)
Crassostrea brasiliana (HQ711627)
Ostrea angelica (KT317130)
Ostrea lurida (NC_022688)
Ostrea permollis (AF052075)
Ostrea puelchana (AF052073)
Ostrea megodon (KX364274)
Ostrea chilensis (JF808186)
Ostrea angasi (AF052063)
Ostrea algoensis (AF052062)
Ostrea denselamellosa (NC_015231)
Ostrea stentina (DQ640402)
Ostrea conchaphila (FJ768528)
Saccostrea glomerata (NC_036483)
Saccostrea echinata (NC_036478)
Saccostrea mytiloides (NC_036479)
Saccostrea kegaki (NC_030533)
Saccostrea malabonensis (KX961677)
Saccostrea palmula (KT317278)
Saccostrea cuccullata (NC_027724)
Saccostrea scyphophilla (NC_013998)
Mytilus edulis (NC_006161)
Mytilus galloprovincialis (NC_006886)
Perna canaliculus (NC_054242)
Mytilus chilensis (NC_030633)
Mytilus platensis (KP100301)
Mytilus unguiculatus (NC_024733)
Mytilus trossulus (NC_007687)
Mytilus californianus (NC_015993)
Perna perna (NC_026288)
Perna viridis (NC_018362)
Conservation
Sequence logo
